# Association of BDNF gene missense polymorphism rs6265 (Val66Met) with three quantitative traits, namely, intelligence quotient, body mass index, and blood pressure: A genetic association analysis from North India

**DOI:** 10.3389/fneur.2022.1035885

**Published:** 2023-01-20

**Authors:** Rafat Fatma, Waseem Chauhan, Mehdi Hayat Shahi, Mohammad Afzal

**Affiliations:** ^1^Human Genetics and Toxicology Laboratory, Section of Genetics, Department of Zoology, Aligarh Muslim University, Aligarh, India; ^2^Interdisciplinary Brain Research Centre, Aligarh Muslim University, Aligarh, India

**Keywords:** BDNF, rs6265, intelligence quotient, body mass index, blood pressure, inheritance models

## Abstract

**Background:**

Brain-derived neurotrophic factor (BDNF), a neurotransmitter modulator, plays a significant role in neuronal survival and growth and participates in neuronal plasticity, thus being essential for learning, memory, and the development of cognition. Additionally, it is crucial for appetite, weight, and metabolic control and plays a pivotal role in the cardiovascular system. The Val66Met polymorphism (rs6265) of the BDNF gene causes a decrease in BDNF secretion and plays a role in impairments in cognition, energy homeostasis, and cardiovascular events. The present study aimed to evaluate the association of polymorphism (rs6265) of the BDNF gene with three quantitative traits simultaneously, namely, intelligence quotient (IQ), body mass index (BMI), and blood pressure (BP).

**Methods:**

Psychometric, morphometric, and physiometric data of the total participants (*N* = 246) were collected. WASI-II^INDIA^ was used to measure cognitive ability. Genotyping was carried out using allele-specific PCR for the rs6265 polymorphism (C196T), and genotypes were determined. Statistical analyses were performed at *p* < 0.05 significance level using MS-Excel and SigmaPlot. The odds ratio models with a 95% confidence interval were used to test the associations. The used models are co-dominant, recessive, dominant, over–dominant, and additive.

**Results:**

The allelic frequencies of alleles C and T were 72 and 28%, respectively. Under the dominant genetic model, a significant susceptible association of minor allele T was observed with a lower average verbal comprehensive index (OR = 2.216, *p* = 0.003, CI (95%) =1.33–3.69), a lower average performance reasoning index (OR = 2.634, *p* < 0.001, CI (95%) = 1.573–4.41), and a lower average full-scale IQ-4 (OR = 3.159, *p* < 0.001, CI (95%) = 1.873–5.328). Carriers of Met-alleles were found to have an increased body mass index (OR = 2.538, *p* < 0.001, CI (95%) = 1.507–4.275), decreased systolic blood pressure (OR = 2.051, *p* = 0.012, CI (95%) = 1.202–3.502), and decreased diastolic blood pressure (OR = 2.162, *p* = 0.006, CI (95%) = 1.278–3.657). Under the recessive genetic model, several folds decrease in IQ and BP and an increase in BMI with the presence of the T allele was also detected.

**Conclusion:**

This novel study may improve our understanding of genetic alterations to the traits and hence be helpful for clinicians and researchers to investigate the diagnostic and prognostic value of this neurotrophic factor.

## 1. Introduction

The BDNF gene on chromosome 11p14.1 in humans encodes brain-derived neurotrophic factor, a member of the neurotrophin family that is extensively expressed in the mammalian brain (1), and it is essential for the survival and differentiation of neuronal populations during development (2, 3). Predominantly, it participates in cognition and memory, including neuroprotection, neuronal and glial growth regulation, and modulation of both short- and long-term synaptic connections (4). It has a higher expression level in the hippocampus, amygdala, cerebellum, and cerebral cortex, with the highest levels shown in hippocampal neurons, whereas in the liver, heart, lung, and blood, it shows lower levels of expression (5, 6). BDNF plays a crucial role in cell differentiation, migration, neuronal survival, dendritic arborization, synaptogenesis, and synaptic plasticity throughout the developmental process (7); consequently, it is involved in learning and memory processes by inducing long-term potentiation in the hippocampus with structural changes in synapses (8).

The BDNF gene has a frequent non-synonymous single-nucleotide polymorphism (SNP), causing methionine (Met) to replace valine (Val) at codon 66 (196 C>T, Val66Met; rs6265), which leads to a decrease in BDNF secretion and causes defects in specific forms of learning (9, 10). Even in heterozygous carriers, it inhibits intracellular trafficking and release of BDNF into the synaptic cleft, which has drawn significant attention to the Val66Met polymorphism in psychiatric studies, and therefore numerous clinical disorders have been studied in relation to the Val66Met polymorphism (11–15). Hence, the Met allele might have pleiotropic consequences as it reduces brain plasticity among those individuals who carry one or two copies of the allele (10). Despite the Met allele being linked to poorer cognitive ability, which signals diminished brain plasticity (the downregulation of synaptic plasticity, particularly in excitatory glutamatergic circuits), it is suggested as a protective factor against mental diseases (16).

Numerous genetic association studies of cognitive performance and a variety of neuropsychiatric illnesses have focused on the BDNF Val66Met polymorphism. To date, a number of studies have shown evidence that this polymorphism impairs hippocampus and cortical function, particularly, which is connected to learning and memory functions; moreover, BDNF concentrations have also been found to be decreased in the brains of mild cognitive impairment patients (10). As a result, a positive correlation was observed between brain BDNF concentration and cognitive performance (7). The polymorphism modulates cognitive capacities in healthy and psychiatric populations, with Met substitution being linked with worse cognitive performance (17–20). In people with obesity and type 2 diabetes, BDNF is downregulated, which has drawn interest in the research on the central regulation of food intake due to its widespread expression in brain regions implicated in appetite regulation (21). It is also important in controlling metabolic processes including fat oxidation and glucose utilization (22–28). The loss of one copy of the BDNF gene in humans is also found to be linked with hyperphagia, severe obesity, reduced cognitive performance, and additionally, poorer locomotor activity (29, 30).

Furthermore, recent studies demonstrated that BDNF and its receptors (tyrosine receptor kinase B, TrkB) are also expressed in the peripheral vasculature, where it stimulates angiogenesis, promotes the survival of endothelial cells, and maintains vascular integrity, therefore, accumulating evidence suggests a pivotal role of BDNF in the cardiovascular system (31–34). Additionally, various studies have reported that the declined BDNF level in plasma, serum, and endothelium was associated with an increased risk of adverse cardiovascular events, blood pressure, and mortality (23, 35–41). On the contrary, high BDNF levels in a large community-based cohort were also prospectively associated with a decreased risk of cardiovascular disease and mortality (14). Furthermore, a study from the South Korean population has suggested that the recessive SNP(Val66Met) rs6265 significantly decreases systolic blood pressure (SBP) (42). Another study has found a positive correlation between blood levels of BDNF and diastolic blood pressure, adipose tissue mass, total cholesterol, low-density lipoprotein (LDL) cholesterol, triglyceride, and body mass index (43).

As discussed above, BDNF plays an important role in cognitive function, the development of intellectual disability, energy homeostasis, caloric intake, and cardiovascular risks; thus, we hypothesized that this particular SNP rs6265 of the BDNF gene must have a significant role in the progression of altered quantitative traits, namely, intelligence quotient (IQ), body mass index (BMI), and blood pressure (BP). We have also proposed the association of BDNF rs6265 polymorphism with IQ, BMI, and BP in our previous report (44). However, no population-based study has been conducted to evaluate the association of the Val66Met polymorphism (rs6265) of the BDNF gene with the aforementioned quantitative traits simultaneously so far. Thus, keeping in view the role of the BDNF gene and its association with these traits, we aimed to genotype the missense SNP rs6265 (196C>T) in our North Indian population to ascertain its link with the selected quantitative traits (IQ, BMI, and BP).

## 2. Materials and methods

### 2.1. Ethical approval and written informed consent

The protocols of this study were reviewed and approved by the Institutional Ethical Committee, Jawaharlal Nehru Medical College (JNMC), Aligarh Muslim University, India. We obtained written informed consent with a signature from each subject included in our study for research and publication.

### 2.2. Study subjects and data collection

This study was conducted in two cities in Uttar Pradesh (the north central zone of India), i.e., Aligarh (the northwest district) and Ambedkar Nagar (the northeast district). A total of 246 healthy participants (aged 6–78 years) were recruited for this study. Subjects who were diseased or not willing to participate in the study were excluded. The psychometric [Verbal Comprehension Index (VCI), Perceptual Reasoning Index (PRI), and Full-Scale Intelligence Quotient-4 (FSIQ-4)], morphometric (height and weight), and physiometric [systolic blood pressure (SBP), diastolic blood pressure (DBP), and pulse rate (PR)] measurements were obtained from each participant.

The VCI, PRI, and FSIQ-4, or general cognitive ability (GCA) were measured using the Wechsler Abbreviated Scale of intelligence, Second Edition, India (WASI-II ^INDIA^) for age groups ranging from 6 to 90 years. It has been adapted from the original WASI developed in the United States. The WASI-II comprises four subtests, i.e., block design, vocabulary, matrix reasoning, and similarities. The vocabulary and similarities subtests compose the VCI, and the block design and matrix reasoning subtests give the PRI. All four subtests included the FSIQ-4 or GCA. Height and weight were taken from the barefooted participants wearing light clothing and measured by using standardized techniques to the nearest 0.1 kg and 0.5 cm, respectively. The body mass index (BMI) was computed as the weight (in kg) and height (in m^2^). Blood pressure (SBP and DBP) in mmHg and PR (bpm) were measured three times by using an “Omron 8712 Automatic Blood Pressure Monitor” after a minimum 5–10 min rest period, and the average of three readings was used in the study. Pulse pressure (PP in mmHg) and mean arterial pressure (MAP in mmHg) were calculated using the formulas, PP = (SBP − DBP) and $\text{MAP = DBP + }\frac{1}{3}(\text{SBP ‒ DBP})$, respectively.

### 2.3. Sample collection and DNA isolation

Blood samples (3 ml) were obtained from the aforementioned individuals by venepuncture from the median cubital vein using a syringe and collected in EDTA-coated (ethylenediaminetetraacetic acid disodium salt) vacutainers; thereafter, DNA isolation was performed for all the samples using the CTAB extraction method (45), and isolated DNA was stored at −20°C till further experimentation.

### 2.4. Primer designing and genotyping of rs6265 polymorphism (C>T)

The freely available online software “OligoCalc” was used for designing the primer for allele-specific polymerase chain reaction (AS-PCR). The sequence of the reverse primer was 5′CAGTTCCACCAGGTGAGAAG3′, and for allele C and allele T, the sequences were 5′CATCCAACAGCTCTTCTATCAC3′ and 5′CATCCAACAGCTCTTCTATCAT3′, respectively. The reverse primer was used with both C and T primers and produced an amplicon size of 241 bp.

Genotyping was carried out using the BIO-RAD T100 Thermal Cycler. A reaction mixture of 10 μl was prepared in a 200 μl PCR tube, containing 1 μl of genomic DNA, 5 μl of master mix, 0.35 μl of each primer, and a final volume was made 10 μl by adding nuclease-free water (NFW). The standardized PCR conditions for BDNF 196C/T included 1 initial cycle of denaturation at 95°C for 5 min, followed by 34 cycles of denaturation at 95°C for 40 s, annealing at 66°C for 40 s, extension at 72°C for 30 s, and final extension at 72°C for 5 min. The amplified products were electrophoresed using 2% agarose gel (Supplementary Figure 1). Three different genotypes were determined in the participants for the BDNF gene rs6265 polymorphism, i.e., CC (Val/Val), CT (Val/Met), and TT (Met/Met).

### 2.5. Statistical analysis

All the analyses were performed using SigmaPlot 11.0 and Microsoft Excel, version 2019. Allelic and genotypic frequencies were calculated by counting the number of alleles, and Hardy-Weinberg equilibrium (HWE) was assessed using the chi-square test for identifying the differences in genotype and allele frequencies between groups. Chi-squared Hardy-Weinberg equilibrium was also tested manually among the group to find out the deviation of the population from the Hardy-Weinberg equilibrium. Quantitative data were presented as mean ± standard deviation (SD), and differences were tested using the Student's t-test and one-way ANOVA. Additionally, the Mann-Whitney Rank Sum test, all pairwise Dunn's test, Bonferroni *t*-test, and Kruskal-Wallis one-way analysis of variance on ranks were applied wherever suitable. Qualitative data were presented as frequency and percentage, and the chi-square test difference was tested between the groups. To find out the relationship between variables, like age and gender with IQ, correlation and regression analyses were performed. The coefficients of correlation (r) and regression (R^2^) were also calculated. The association of polymorphism and traits were estimated by odds ratios (ORs), using co-dominant, recessive, dominant, over-dominant, and additive models of inheritance, at 95% confidence intervals (CI) between the groups for those holding the mutant alleles (46). Statistical significance was defined at the standard 5% level (*p* < 0.05).

For the traits recorded, all the participants were categorized into two groups. For the total population surveyed, the mean values for VCI, PRI, and FSIQ-4 were 69.56, 81.38, and 75.19, respectively (Table 1). We further grouped the individuals into the “below average category (BA)” and “average category (A)” for having their IQ values below, and equal to, or above the mean values, respectively. The normal range of BMI was considered 18.5–22.9 kg/m^2^ (47, 48), so the individuals were categorized into “increased BMI (IBMI)” and “normal BMI (NBMI)” with BMI > 22.9 and BMI ≤ 22.9, respectively, for additional analysis. SBP (120 mmHg) and DBP (80 mmHg) were considered normal, and the individuals were categorized into “decreased (D)” and “normal (N)” according to their blood pressure readings. Hence, to find out the association of polymorphism rs6265 with the traits in question, we merged two categories to obtain better results.

**Table 1:** Characteristics of studied participants (*N* = 246) in terms of demographic, psychometric, morphometric, and physiometric data.

**Attributes**	**Total (*N* = 246)**	**Male (*n* = 138)**	**Female (*n* = 108)**	***P-*value**
Age (Years)	30.37 ± 12.72	30.55 ± 12.46	30.13 ± 13.09	0.598
Genotype CC (*n*)	127	78	49	
Genotype CT (*n*)	98	52	46	
Genotype TT (*n*)	21	8	13	0.1007
VCI	69.56 ± 16.48	71.36 ± 16.26	67.27 ± 16.57	0.044^*^
PRI	81.38 ± 18.90	84.52 ± 18.19	77.37 ± 19.12	0.001^*^
FSIQ-4	75.19 ± 16.53	77.86 ± 16.02	71.77 ± 16.61	0.001^*^
Height (cm)	160.96 ± 11.68	163.89 ± 11.54	157.22 ± 10.79	< 0.001^*^
Weight (kg)	59.57 ± 12.27	60.17 ± 12.68	58.80 ± 11.75	0.485
BMI (kg/m^2^)	22.86 ± 3.96	22.24 ± 3.92	23.65 ± 3.90	0.003^*^
SBP (mmHg)	124.11 ± 12.61	125.66 ± 12.04	122.13 ± 13.09	0.013^*^
DBP (mmHg)	82.08 ± 8.25	82.99 ± 7.59	80.93 ± 8.92	0.036^*^
PR (bpm)	83.94 ± 11.99	83.78 ± 11.32	84.16 ± 12.86	0.696
PP (mmHg)	42.02 ± 9.78	42.67 ± 9.52	41.19 ± 10.07	0.309
MAP (mmHg)	96.09 ± 8.78	97.22 ± 8.16	94.66 ± 9.35	0.007^*^

Data presented as mean ± standard deviation, ^*^statistically significant at p < 0.05, chi-square test and Student's t-test followed by Mann-Whitney Rank Sum test was applied between male and female. n, number; VCI, verbal comprehensive index; PRI, perceptual reasoning index; FSIQ-4, full scale intelligence quotient-4; BMI, body mass index; SBP (mmHg), systolic blood pressure (millimeters of mercury); DBP, diastolic blood pressure; PR, pulse rate; bpm, beats per minute; PP, pulse pressure; MAP, mean arterial pressure.

## 3. Results

### 3.1. Demographic characteristics of the population surveyed

A total of 246 healthy individuals were included in this study from North Indian regions (Aligarh and Ambedkar Nagar). Out of the total population, approximately 56% were men and 44% were women (Table 1). The mean age of the participants involved in the study was 30.37 ± 12.72 (years), however the age differences between men and women show a non-significant difference in mean values. Maximum proportions of the population surveyed were from sub-urban areas and socioeconomically belonged to the middle class; however, few of them were upper and lower classes also. The localities of the surveyed area comprised different castes like Ashraf (upper caste, e.g., Syed, Sheikh, Pathan, and Rajpoot) and Ajlaf (lower caste, e.g., Quraishi and Ansari). The individuals included in the study were moderately educated, although men were slightly more educated than women. Distribution of genotypes (CC, CT, and TT) among the studied population showed non-significant differences with gender (*p* = 0.1007). Student's *t*-test followed by Mann-Whitney Rank Sum test revealed that male participants were having significantly higher cognitive values, i.e., VCI, PRI, and FSIQ-4 (*p* = 0.044, 0.001, and 0.001, respectively) than female participants (Table 1). Regression and correlation analyses of IQ with age and gender showed low values of coefficients, i.e., for FSIQ-4, R^2^ = 0.021, r = −0.14 with age, R^2^ = 0.0057, r = −0.075 with men, and R^2^ = 0.0484, r = −0.22 with women (Supplementary Figures 2–4). In terms of morphometric parameters, the height (cm) of the men was also significantly higher (*p* < 0.001) when compared to women, but weight showed non-significant differences between the two genders, although BMI (kg/m^2^) values observed were greater in the case of women as compared to men (*p* = 0.003). The physiometric traits recorded during the survey also showed significant differences in the cases of SBP, DBP, and MAP, with *p* = 0.013, 0.036, and 0.007, respectively, but non-significant results were observed for PR and PP (Table 1).

### 3.2. Genotypic and allelic frequency of rs6265 polymorphism in BDNF gene

By genotyping the individuals using allele-specific PCR, it was found that 51.63% individuals of the total population (surveyed) had a CC genotype, 39.84% had a CT genotype, and only 8.53% had a TT genotype (Supplementary Table 1). The genotypic and allelic frequencies were calculated by gene counting and shown in Supplementary Table 1. The allelic frequency for allele C, i.e., f _(C)_ was found to be 0.72 (major allele) and f _(T)_ was 0.28 (minor allele) for allele T. Chi-squared Hardy-Weinberg Equilibrium analysis for the particular gene revealed non-significant differences (*p* > 0.064), suggesting that the studied population did not deviate from Hardy-Weinberg Equilibrium, meaning that the particular population was in equilibrium for rs6265 polymorphism of BDNF gene (Supplementary Table 2).

### 3.3. Psychometric traits and BDNF gene polymorphism rs6265 (C>T)

**Figure 1:**
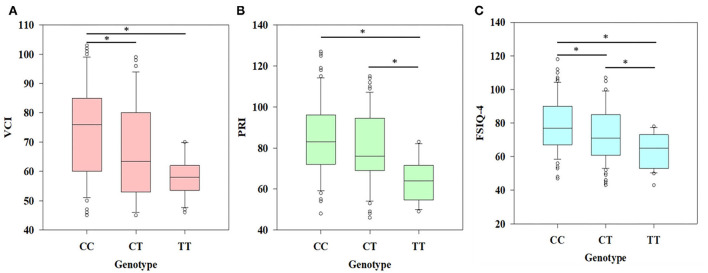
Association of different genotypes (CC, CT, and TT) and psychometric traits shown by the results of ANOVA. **(A)** Significant difference (*p* < 0.001) was observed in the case of VCI (verbal comprehensive index) between CC and TT genotype and CC and CT genotype, **(B)** significant difference (*p* < 0.001) was observed in the case of PRI (perceptual reasoning index) between CC and TT genotype and CT and TT genotype, and **(C)** FSIQ-4 (full scale intelligence quotient-4) showing significant differences (*p* < 0.001) among all three genotypes (^*^p < 0.05).

By applying one-way ANOVA followed by all pairwise Dunn's test for cognitive values (VCI, PRI, and FSIQ-4) among groups with different genotypes (CC, CT, and TT), statistically significant (*p* < 0.001) results were obtained among the groups (Figure 1). In the case of VCI, the frequency of the rs6265 T allele among BAVCI individuals (36.51%) was significantly higher than among AVCI individuals (20 %) with a *p*-value of <0.001 (Table 2). The statistical analysis odds ratio revealed a significant association between the polymorphism rs6265 and BAVCI in recessive (OR = 10.477, *p* < 0.001, CI (95%) = 2.384–46.038) and dominant (OR = 2.216, *p* = 0.003, CI (95%) = 1.330–3.691) models of inheritance (Table 2). The other models were sufficiently different from these models, did not produce a good fit, and could be rejected (Table 2). Similarly, individuals with BAPRI showed a significantly higher value of T allele frequency (37.40%) as compared to APRI individuals (*p* < 0.001) (Table 3). In this case, the recessive model (OR = 10.292, *p* < 0.001, CI (95%) = 2.342–45.225) and the dominant model (OR = 2.634, *p* < 0.001, CI (95%) =1.573–4.410) both showed a significant association of BARPI with rs6265 polymorphism of BDNF gene (Table 3). A similar trend was observed, as the allelic frequency of T was significantly higher (38.08%) in BAFSIQ-4 individuals compared with AFSIQ-4 ones (17.67%) with a *p*-value of < 0.001 (Table 4). Like VCI and PRI, FSIQ-4 also showed a significant association of polymorphism rs6265 with BAFSIQ-4 in both recessive and dominant models of inheritance (OR = 9.757, *p* <0.001, CI (95%) = 2.220–42.873 and OR = 3.159, *p* < 0.001, CI (95%) = 1.873–5.328, respectively). Other suggested models could be rejected because of producing no good results (Table 4).

**Table 2:** BDNF gene polymorphism under co-dominant, recessive, dominant, over-dominant, and additive models in individuals with below average VCI (BAVCI) and average VCI (AVCI).

**Genotype/Allele**	**BAVCI** **(*****n*** = **126)**		**AVCI** **(*****n*** = **120)**		**χ^2^**	***P*-value**	**OR**	**CI (95%)**
	**No**.	**%**	**No**.	**%**				
**Co-dominant model**								
CC^®^	53	42.06	74	61.67				
CT	54	42.86	44	36.66	3.447	0.063	0.584	0.343–0.993
TT	19	15.08	02	1.67	15.243	< 0.001	0.0754	0.0164–0.338
**Recessive model**								
TT	19	15.08	02	1.67				
CC+CT	107	84.92	118	98.33	12.496	< 0.001^*^	10.477	2.384–46.038
**Dominant model**								
TT+CT	73	57.94	46	38.33				
CC	53	42.06	74	61.67	8.689	0.003^*^	2.216	1.330–3.691
**Over-dominant model**								
CT	54	42.86	44	36.66				
CC+TT	72	57.14	76	63.34	0.741	0.389	1.295	0.776–2.162
**Additive model**								
C	160	63.49	192	80				
T	92	36.51	48	20	15.654	< 0.001^*^	0.435	0.289–0.653

^®^, Reference; ^*^, significant value; No, number; OR, odds ratio; CI, confidence interval.

**Table 3:** BDNF gene polymorphism under co-dominant, recessive, dominant, over-dominant, and additive models in individuals below average PRI (BAPRI) and average PRI (APRI).

**Genotype/Allele**	**BAPRI** **(*****n*** = **127)**		**APRI** **(*****n*** = **119)**		**χ^2^**	***P*-value**	**OR**	**CI (95%)**
	**No**.	**%**	**No**.	**%**				
**Co-dominant model**								
CC^®^	51	40.16	76	63.87				
CT	57	44.88	41	34.45	6.482	0.011^*^	0.483	0.283–0.825
TT	19	14.96	02	1.68	16.341	< 0.001^*^	0.0706	0.0158–0.316
**Recessive model**								
TT	19	14.96	02	1.68				
CC+CT	108	85.04	117	98.32	12.228	< 0.001^*^	10.292	2.342–45.225
**Dominant model**								
TT+CT	76	59.84	43	36.13				
CC	51	40.16	76	63.87	12.894	< 0.001^*^	2.634	1.573–4.410
**Over-dominant model**								
CT	57	44.88	41	34.45				
CC+TT	70	55.12	78	65.55	2.369	0.124	1.549	0.926–2.593
**Additive model**								
C	159	62.60	193	81.09				
T	95	37.40	45	18.91	19.744	< 0.001^*^	0.390	0.258–0.589

^®^, Reference; ^*^, significant value; OR, odds ratio; CI, confidence interval.

**Table 4:** BDNF gene polymorphism under co-dominant, recessive, dominant, over-dominant, and additive models in individuals with below average FSIQ-4 (BAFSIQ-4) and average FSIQ-4 (AFSIQ-4).

**Genotype/Allele**	**BAFSIQ-4** **(*****n*** = **130)**		**AFSIQ-4** **(*****n*** = **116)**		**χ^2^**	***P*-value**	**OR**	**CI (95%)**
	**No**.	**%**	**No**.	**%**				
**Co-dominant model**								
CC^®^	50	38.46	77	66.38				
CT	61	46.92	37	31.89	10.683	0.001^*^	0.394	0.229–0.677
TT	19	14.62	02	1.73	16.915	< 0.001^*^	0.0684	0.0153–0.306
**Recessive model**								
TT	19	14.62	02	1.73				
CC+CT	111	85.38	114	98.27	11.449	< 0.001^*^	9.757	2.220–42.873
**Dominant model**								
TT+CT	80	61.54	39	33.62				
CC	50	38.46	77	66.38	18.030	< 0.001^*^	3.159	1.873–5.328
**Over-dominant model**								
CT	61	46.92	37	31.89				
CC+TT	69	53.08	79	68.11	5.165	0.023^*^	1.888	1.21–3.177
**Additive model**								
C	161	61.92	191	82.33				
T	99	38.08	41	17.67	24.081	< 0.001^*^	0.349	0.229–0.531

^®^, Reference; ^*^, significant value; OR, odds ratio; CI, confidence interval.

### 3.4. Morphometric traits and BDNF gene polymorphism rs6265 (C>T)

**Figure 2:**
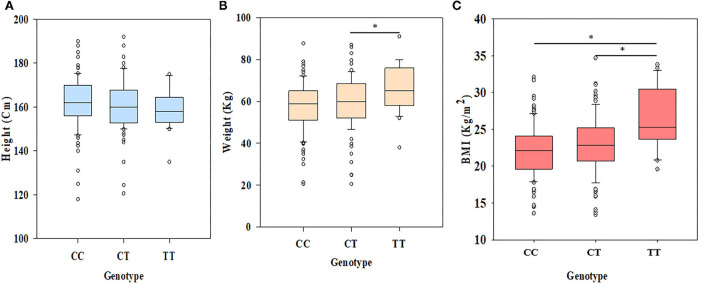
Results of ANOVA among different genotypes for morphometric traits. **(A)** No significant difference (*p* = 0.172) was observed in the case of height among genotypes, but **(B)** showing significant difference (*p* = 0.015) for weight between CT and TT, and **(C)** BMI between CC and TT (*p* < 0.001) and CT and TT (*p* = 0.002), respectively (^*^p < 0.05).

Kruskal-Wallis one-way analysis of variance on ranks application on height (Cm) of different genotype categories revealed no significant association among the groups (*p* = 0.172) (Figure 2A), while significant difference (*p* = 0.015) was observed in the case of weight (kg) among different genotype categories after applying Dunn's method (Figure 2B). One-way ANOVA followed by the Bonferroni t-test on BMI values revealed a statistically significant (*p* < 0.001) difference among different groups (Figure 2C). The values of significance were found to be different among different groups for BMI. The individuals with CC homozygous (Val/Val) and TT homozygous (Met/Met) showed a highly significant difference with a *p-*value of <0.001, but between CT heterozygous (Val/Met) and TT homozygous (Met/Met), it was found to be significant at only *p* = 0.002 (Figure 2C). Further analysis of two BMI categories (IBMI and NBMI) with respect to genotypes showed a significant association of the rs6265 polymorphism with IBMI in the recessive (OR=5.172, *p*=0.002, CI (95%) =1.829-14.627) and dominant (OR = 2.538, *p* < 0.001, CI (95%) = 1.507–4.275) models of inheritance (Table 5). The minor allele T showed higher frequency (38.37%) in the case of the increased BMI category as compared to Normal BMI (21.18%) with a *p*-value of <0.001 (Table 5). Other models of inheritance did not produce good results and were therefore rejected.

**Table 5:** BDNF gene polymorphism under co-dominant, recessive, dominant, over-dominant, and additive models in individuals with increased BMI (IBMI) and normal BMI (NBMI).

**Genotype/Allele**	**IBMI** **(*****n*** = **102)**		**NBMI** **(*****n*** = **144)**		**χ^2^**	***P*-value**	**OR**	**CI (95%)**
	**No**.	**%**	**No**.	**%**				
**Co-dominant model**								
CC^®^	39	38.23	88	61.11				
CT	47	46.08	51	35.42	6.260	0.012^*^	0.481	0.278–0.831
TT	16	15.68	05	3.47	14.075	< 0.001^*^	0.138	0.0474–0.405
**Recessive model**								
TT	16	15.68	05	3.47				
CC+CT	86	84.32	139	96.53	9.897	0.002^*^	5.172	1.829–14.627
**Dominant model**								
TT+CT	63	61.77	56	38.89				
CC	39	38.23	88	61.11	11.612	< 0.001^*^	2.538	1.507–4.275
**Over-dominant model**								
CT	47	46.08	51	35.42				
CC+TT	55	53.92	93	64.58	2.404	0.121	1.588	0.928–2.616
**Additive model**								
C	125	61.27	227	78.82				
T	79	38.73	61	21.18	17.204	< 0.001^*^	0.425	0.285–0.634

^®^, Reference; ^*^, significant value; OR, odds ratio; CI, confidence interval.

### 3.5. Physiometric traits and BDNF gene polymorphism rs6265 (C>T)

**Figure 3:**
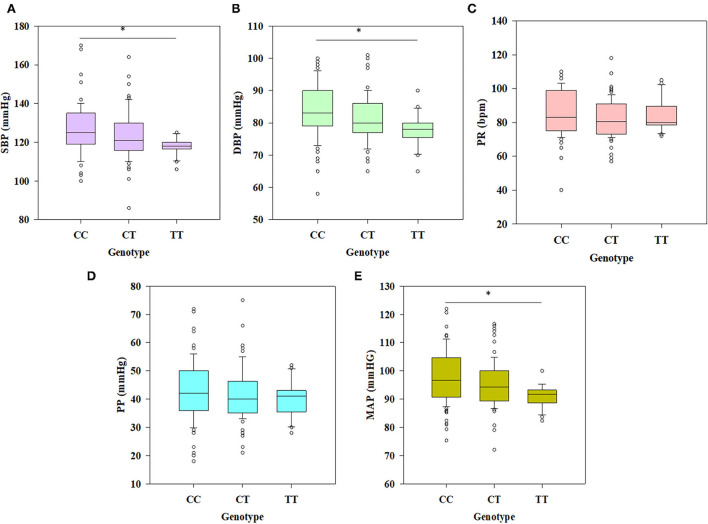
Box plots deciphering the results of ANOVA among different genotypes (CC, CT, and TT) for physiometric traits. **(A, B, E)** Significant differences were observed between genotypes CC and TT with SBP (*p* = 0.001), DBP (*p* = 0.001), and MAP (*p* = 0.001); **(C, D)** non-significant differences were observed for PR (*p* = 0.087) and PP (*p* = 0.402), respectively (^*^p < 0.05).

Statistically significant differences were observed in SBP (*p* = 0.001), DBP (*p* = 0.01), and MAP (*p* = 0.001) values among different genotypes (CC, CT, and TT) after applying one-way ANOVA followed by all pairwise Dunn's test (Figures 3A, B, E). Kruskal-Wallis one-way analysis of variance on ranks revealed that PR (*p* = 0.087) and PP (*p* = 0.402) values had non-significant differences among the groups (CC, CT, and TT), which is clear from Figures 3C, D. Further statistical analyses were also conducted to test the association of decreased SBP and DBP with the polymorphism rs6265 in individuals having different genotypes. Among all the models tested for significance, the recessive model of inheritance in SBP (OR = 4.338, *p* = 0.003, CI (95%) = 1.678–11.215) and DBP (OR = 3.696, *p* = 0.009, CI (95%) = 1.433–9.35) showed a significant association of T allele with a decrease in their values (Tables 6, 7). Dominant models also showed the same results in both SBP and DBP having OR = 2.051, *p* = 0.012, CI (95%) = 1.202–3.502 and OR = 2.162, *p*=0.006, CI (95%) = 1.278–3.657, respectively (Tables 6, 7). The frequency of the minor allele (T) was higher in the cases of decreased SBP (38.34%) and DBP (37.64%) as compared with normal values of these parameters (*p* = 0.010 and *p* < 0.001, respectively) (Tables 6, 7).

**Table 6:** BDNF gene polymorphism under co-dominant, recessive, dominant, over-dominant, and additive models in individuals with decreased SBP (DSBP) and normal SBP (NSBP).

**Genotype/Allele**	**DSBP** **(*****n*** = **85)**		**NSBP** **(*****n*** = **161)**		**χ^2^**	***P*-value**	**OR**	**CI (95%)**
	**No**.	**%**	**No**.	**%**				
**Co-dominant model**								
CC^®^	34	40	93	57.76				
CT	37	43.53	61	37.89	2.602	0.107	0.603	0.342–1.062
TT	14	16.47	07	4.35	11.331	< 0.001^*^	0.183	0.0680–0.491
**Recessive model**								
TT	14	16.47	07	4.35				
CC+CT	71	83.53	154	95.65	8.976	0.003^*^	4.338	1.678–11.215
**Dominant model**								
TT+CT	51	60	68	42.24				
CC	34	40	93	57.76	6.336	0.012^*^	2.051	1.202–3.502
**Over-dominant model**								
CT	37	43.53	61	37.89				
CC+TT	48	56.47	100	62.11	0.522	0.470	1.264	0.741–2.156
**Additive model**								
C	105	61.76	247	76.70				
T	65	38.34	75	23.30	6.685	0.010^*^	1.864	1.184–2.935

^®^, Reference; ^*^, significant value; OR, odds ratio; CI, confidence interval.

**Table 7:** BDNF gene polymorphism under co-dominant, recessive, dominant, over-dominant, and additive models in individuals with decreased DBP (DDBP) and normal DBP (NDBP).

**Genotype/Allele**	**DDBP** **(*****n*** = **93)**		**NDBP** **(*****n*** = **153)**		**χ^2^**	***P*-value**	**OR**	**CI (95%)**
	**No**.	**%**	**No**.	**%**				
**Co-dominant model**								
CC^®^	37	39.79	90	58.82				
CT	42	45.16	56	36.60	3.990	0.046^*^	0.584	0.315–0.954
TT	14	15.05	07	4.58	9.640	0.002^*^	0.206	0.0768–0.550
**Recessive model**								
TT	14	15.05	07	4.58				
CC+CT	79	84.95	146	95.42	6.847	0.009^*^	3.696	1.433–9.35
**Dominant model**								
TT+CT	56	60.21	63	41.18				
CC	37	39.79	90	58.82	7.650	0.006^*^	2.162	1.278–3.657
**Over-dominant model**								
CT	42	45.16	56	36.60				
CC+TT	51	54.84	97	63.40	1.429	0.232	1.426	0.844–2.410
**Additive model**								
C	116	63.36	236	77.13				
T	70	37.64	70	22.87	11.663	< 0.001^*^	0.492	0.330–0.732

^®^, Reference; ^*^, significant value; OR, odds ratio; CI, confidence interval.

## 4. Discussion

To evaluate the association of Val66Met polymorphism (rs6265) of the BDNF gene with IQ (VCI, PRI, and FSIQ-4), BMI, and BP (SBP and DBP) concomitantly, we incorporated 246 individuals, comprising 56% men and 44% women (Table 1), and collected their data and blood samples after obtaining written informed consents. After genotyping analysis of the Val66Met polymorphism (C>T), we found that the allelic frequency of the minor allele (T) was 0.28 and the major allele (C) was 0.72 (Supplementary Table 1). Approximately similar results were obtained from the study (49), also for south Asian and global populations previously published in databases (dbSNP, GnomAD_exome, 1000G, dbGaP).

There is mixed research suggesting gender-based differences in cognitive ability among men and women; similarly, our results also showed a significant difference in cognitive values between men and women (Table 1). This difference might be due to the influence of the environment and other genes or may be due to sex hormones (50–52). Although regression and correlation analyses of IQ (VCI, PRI, and FSIQ-4) with respect to age and gender revealed non-significant results, suggesting that there was no age- or gender-specific difference in the population surveyed (Supplementary Figures 2–4). So, age and gender were not considered confounding variables in this case; thus, this aspect was not followed up in further analysis. Men and women in this study also showed significant differences in height and BMI (Table 1); height in men tends to be higher as compared to that in women, but BMI was found to be higher in women than men (53). For physiometric traits, male participants showed significantly higher blood pressure (SBP, DBP, and MAP) compared to female participants (Table 1), as also suggested by findings of studies from other populations worldwide (54–56). This difference in blood pressure is due to the protective nature of some biological and behavioral factors against hypertension in women. These factors include sex hormones, chromosomal differences, and other biological sex differences (57).

Various studies have reported the association of the missense SNP rs6265 (Val66Met) of the BDNF gene with brain development, cognition, learning, memory, numerous clinical disorders, food intake, appetite regulation, cardiovascular risks, blood pressure, and mortality (10, 15, 22, 37). Thus, BDNF, the most prevalent growth factor in the central nervous system, serves as a potential marker for psychiatric diseases and mental illnesses and is widely implicated in schizophrenia, major depressive disorder (MDD), and bipolar disorder (58–63). This SNP (rs6265) is responsible for the downregulation of the BDNF gene, causing decreased BDNF concentration and hence affecting many quantitative traits like psychometric, morphometric, and physiometric traits. Our work indicates that individuals with Met/Met homozygosity have below-average verbal IQ (VCI) or higher verbal recall errors, but Val/Val and Val/Met have average values for the same, supported by the fact that there is a decrease in gray matter volume of the hippocampus in BDNF Met allele carriers compared to BDNF Val/Val individuals as reported earlier (10). Since, the frequency of allele T was higher in individuals having BAVCI (36.51%) than for AVCI (20%) as mentioned earlier, therefore, using recessive and dominant genetic models, we analyzed that this polymorphism confers 10.477 and 2.216-fold increased risk for the development of lower VCI values, respectively (Table 2). Similarly, after implementing the recessive and dominant genetic model of inheritance for the association analysis of PRI, it could be inferred that minor allele T is responsible for 10.292- and 2.634-fold increased risk of a lower PRI, respectively. Hence, participants having a TT or Met/Met homozygous state have a significantly lower PRI as compared with CT (Val/Met) heterozygous and CC (Val/Val) homozygous individuals. The frequency of the minor allele was higher in individuals having significantly lower PRI values (37.40 %) than in normal PRI (18.91%) individuals (Table 3). These results are consistent with a recent study, where scientists found a significant positive correlation (r = 0.424 and *p* = 0.001) between BDNF level and performance IQ (PRI) (64).

Likewise, FSIQ-4 or GCA or “g” also shows an association of the minor allele T with 9.757- and 3.159-fold increased risk of below-average IQ under both recessive and dominant models of inheritance (Table 4). So far, a number of studies have advised that the BDNF Val66Met polymorphism leads to impairments in hippocampal and cortical function, which are specifically related to learning and memory processes and lead to worse cognitive performance in healthy individuals and psychiatric populations (17, 19, 20). Our investigation also goes with the trend that Met/Met individuals have below-average IQ than the other two groups (10). The allelic frequency of the minor allele (T) is found to be significantly higher (38.08%) values among individuals having below average IQ as compared to the other group (Table 4), supporting the association of the T allele in lowering down the cognitive ability of individuals. Similar studies also reported the association of this polymorphism with poor episodic memory, abnormal hippocampal activation, abnormal intracellular trafficking and dysregulation of BDNF secretion, poorer neurocognitive function, worse cognitive performance, especially in overall intelligence, verbal comprehension, and perceptual reasoning (13, 65, 66).

Evidence suggests that BDNF may play a role in appetite, weight, and metabolic control since carriers of the Val66Met BDNF Met-alleles showed substantially greater C-reactive protein and calorie intake in the form of fat and protein compared to carriers of the Val-allele hence leading to an increase in BMI (22). Our study produces analogous results, i.e., 5.172- and 2.538-fold increased BMI observed in Met/Met individuals as compared to Met/Val and Val/Val individuals under the recessive and dominant model of inheritance, respectively (Table 5). Higher allelic frequency (38.73 %) of the minor allele (T) in the case of individuals with increased BMI than normal BMI individuals showed statistical significance (*p* < 0.001). Numerous studies have recommended the role of BDNF (rs6265) in the progression of obesity or a significant increase in BMI. Lower levels of circulating BDNF increase food intake, weight gain, and adiposity, and hence can be considered a genetic determinant of obesity or IBMI (67–71).

Despite its role in cognition, memory, clinical disorders, and appetite regulation, BDNF is responsible for the alteration of some physiometric traits and appeared to be involved in the progression of altered events in the cardiovascular system, hence affecting the BP among individuals (15, 37, 40, 41). Our present investigation revealed a significant positive association of a decrease in blood pressure (SBP and DBP) with Met/Met under recessive (OR = 4.338 and OR = 3.696, respectively) and dominant (OR = 2.051 and OR = 2.162, respectively) models of inheritance (Tables 6, 7). Our results are in concordance with previous studies, reporting a decline in SBP with decreasing BDNF level due to the homozygous T allele (TT genotype or Met/Met) (42), and a decrease in DBP due to the Met/Met homozygous condition (43). The allelic frequency of minor allele T was found to be significantly higher in individuals having lower values of SBP (38.34%) and DBP (37.64%) than in individuals with normal blood pressure (Tables 6, 7). Hence, an association between low levels of BDNF in the serum and an increase in cardiovascular risk, severe cardiac diseases, coronary heart disease (CHD), and the growth factor is seen, which directs the response of the cardiovascular system to acute and chronic injury (32, 72–74).

## 5. Conclusion

This is a novel investigation linking the BDNF rs6265 (Val66Met, C196T) polymorphism with a range of quantitative traits in the North Indian population. We genotyped 246 individuals from two North Indian districts and recorded their parameters related to psychometric, morphometric, and physiometric traits using standard measures. We observed a significant association between carriers of the Met-alleles with a decrease in cognitive ability (VCI, PRI, and FSIQ-4), an increase in BMI, and a decrease in blood pressure (SBP and DBP), as compared to Val-allele carriers. We also noted an increased frequency of allele T in the individuals with altered values of the nominated traits. Hence, we suggest that the BDNF polymorphism rs6265 can be used as an early biomarker for the identification of intellectual disability, predisposition to obesity, and increased risk for cardiovascular events. However, future research is desirable to endorse our conclusions and determine whether carriers of this BDNF polymorphism are genetically predisposed to increased energy intake, weight gain, and metabolic complications. In the era of personalized medicine, this evidence would be crucial to advise early intervention tactics intended to improve cognitive power, optimize obesity, and realize chronic cardiovascular disease prevention and management.

## Data availability statement

The raw data supporting the conclusions of this article will be made available by the authors, without undue reservation.

## Ethics statement

The studies involving human participants were reviewed and approved by Institutional Ethics Committee, JNMC, AMU, Aligarh. Written informed consent to participate in this study was provided by the participants on their own or by their legal guardian/next of kin.

## Author contributions

RF: conceptualization, study design, data curation, data analysis, investigation, methodology, resources, original draft, and writing–review and editing. WC: data analysis, investigation, conceptualization, and writing–review and editing. MHS: validation, formal analysis, and writing–review and editing. MA: supervision, study design, validation, visualization, conceptualization, and formal analysis. All authors contributed to the article and approved the submitted version.
