# Supplementary material for: Association of BDNF gene missense polymorphism rs6265 (Val66Met) with three quantitative traits, namely, intelligence quotient, body mass index, and blood pressure: A genetic association analysis from North India
Source: Front Neurol. 2023 Jan 20;13:1035885. doi: 10.3389/fneur.2022.1035885 (PMC9894895; doi:10.3389/fneur.2022.1035885)
Supplement: Supplementary file 1 [file Table_1.DOCX]

**Supplementary Table 1**: Distribution of genotypes among the surveyed population along with their genotypic and allelic frequencies.

| **Genotype** | **No. of individuals** | **Percentage (%)** | **Genotypic frequency** | **Allelic frequency** |
| --- | --- | --- | --- | --- |
| **CC** | 127 | 51.63 | f _(CC)_ = 0.5184 | f _(C)_ = 0.72  f _(T)_ = 0.28 |
| **CT** | 98 | 39.84 | f _(CT)_ = 0.0784 |  |
| **TT** | 21 | 8.53 | f _(TT)_ = 0.4032 |  |

**Supplementary Table 2**: Chi-squared Hardy-Weinberg Equilibrium for the population surveyed showing non-significant difference at *p* < 0.05.

| **Genotype** | **Observed** | **Expected** | **df** | **χ^2^** | ***P*-value** |
| --- | --- | --- | --- | --- | --- |
| **CC** | 127 | 127.53 | 1 | 0.1696 | >0.064 |
| **CT** | 98 | 99.18 |  |  |  |
| **TT** | 21 | 19.28 |  |  |  |


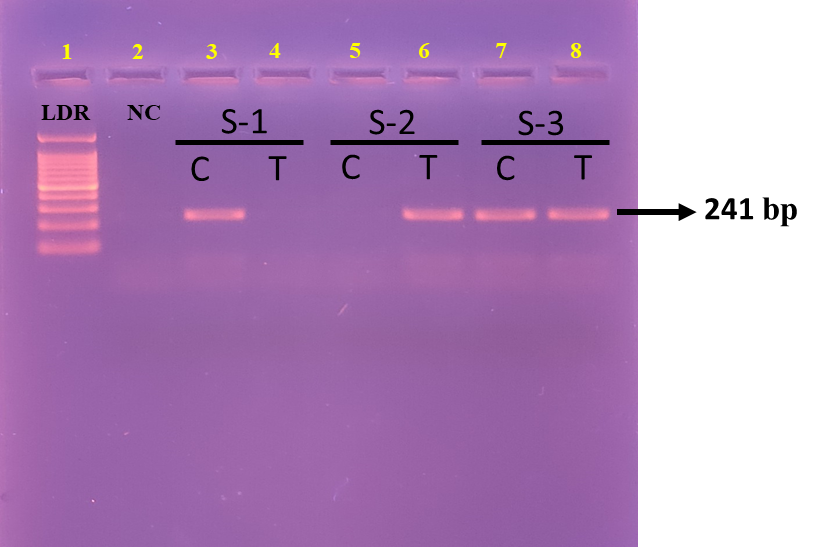


**Supplementary Figure 1**: Gel image showing results of Allele-specific PCR for the detection of rs6265 polymorphism of BDNF gene. Size of the amplicon is 241 base pairs. Lane 1 showing ladder 100bp (1500-100bp), Lane 2 showing negative control without DNA sample, Lane 3 and 4 showing homozygous state for CC in subject 1, where primer for T allele not showing band, Lane 5 and 6 showing homozygous state for TT in subject 2, where primer for C allele not showing band, Lane 7 and 8 showing heterozygous state (CT) in subject 3, here, primer for both alleles (T and C) showing band of 241bp. (LDR= ladder, NC= negative control, S-1=subject 1, S-2= subject 2, S-3=subject 3).


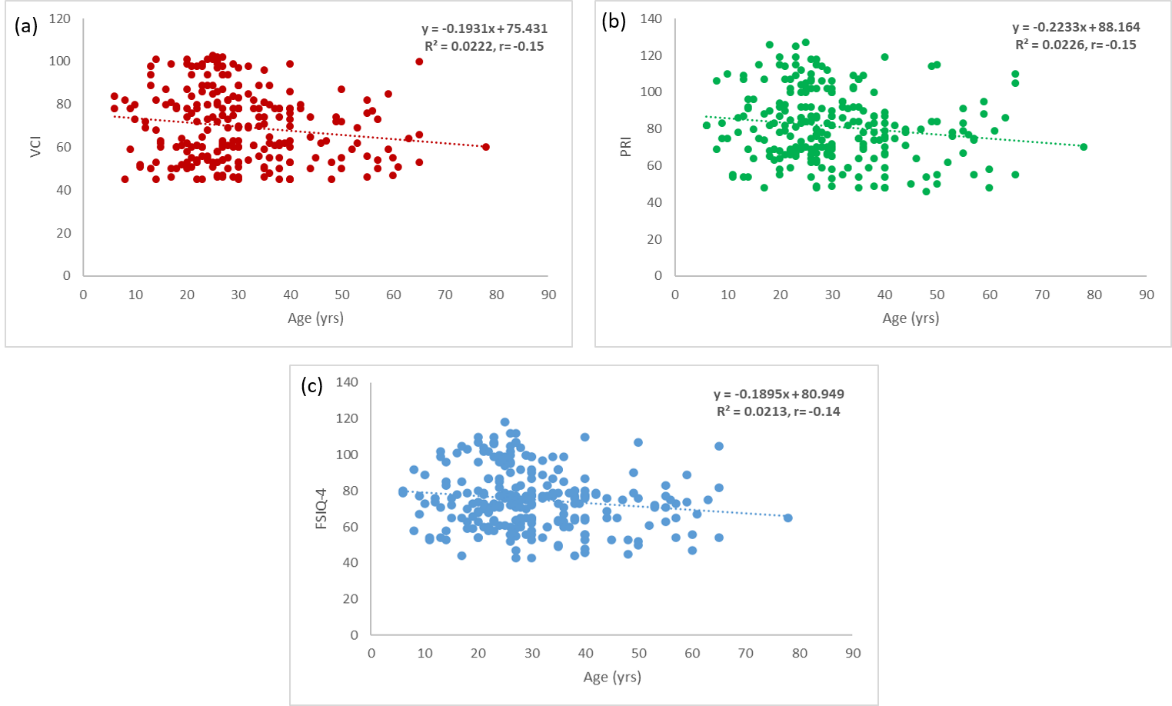


**Supplementary Figure 2:** Regression and correlation analysis of IQ with respect to age of the participants, regression and correlation coefficients (R^2^ and r), respectively, showing lower and non-significant values, suggesting no correlation or dependency between these two variables. With respect to age the coefficients are shown (a) for VCI, R^2^ = 0.022 and r= -0.15, (b) for PRI, R^2^= 0.23 and r= -0.15, (c) for FSIQ-4, R^2^= 0.021 and r= -0.14.


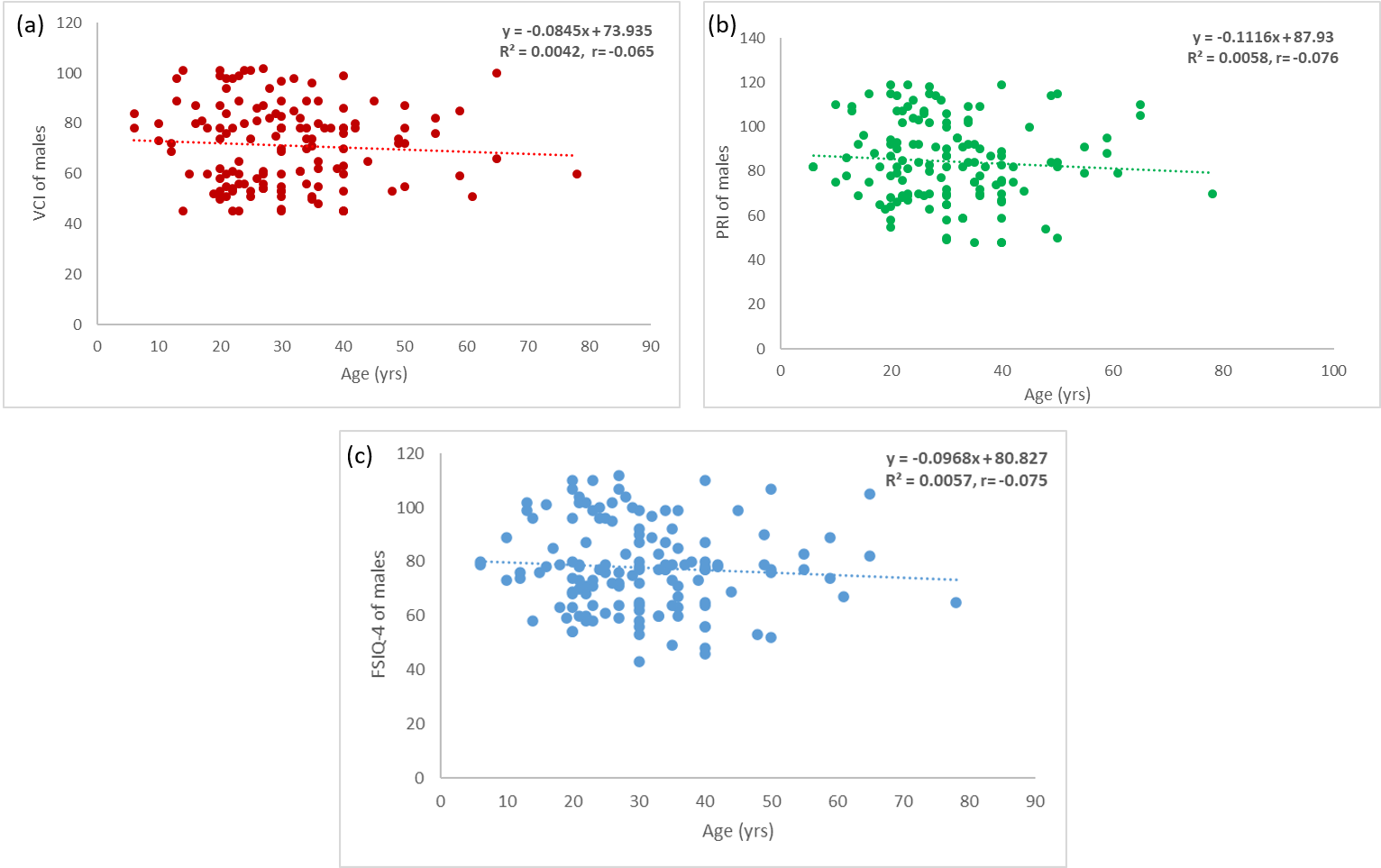


**Supplementary Figure 3:** Relationship of male gender with IQ among the participants using regression and correlation analysis with regression and correlation coefficients (R^2^ and r), respectively, showing lower and non-significant values, suggesting no correlation or dependency between gender and IQ. With respect to male gender the coefficients are shown (a) for VCI, R^2^ = 0.0042 and r= -0.065, (b) for PRI, R^2^= 0.0058 and r= -0.076, (c) for FSIQ-4, R^2^= 0.0057 and r= -0.075.


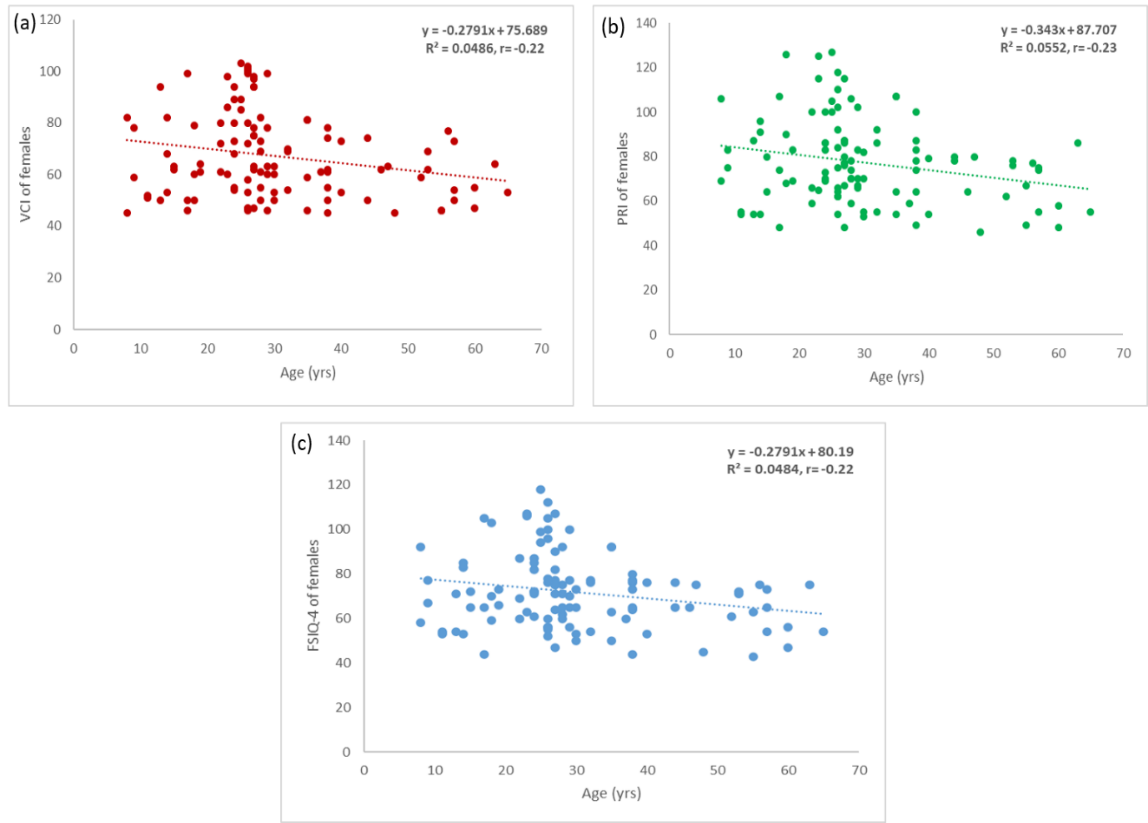


**Supplementary Figure 4:** Regression and correlation analysis of IQ with respect to female participants, regression and correlation coefficients (R^2^ and r), respectively, showing lower and non-significant values, suggesting no correlation or dependency between these two variables. With respect to age of females the coefficients are shown (a) for VCI, R^2^ = 0.0486 and r= -0.22, (b) for PRI, R^2^= 0.0552 and r= -0.23, (c) for FSIQ-4, R^2^= 0.048 and r= -0.22.
